# Histopathological characterisation of retinal lesions associated to *Diplostomum* species (Platyhelminthes: Trematoda) infection in polymorphic Arctic charr *Salvelinus alpinus*

**DOI:** 10.1016/j.ijppaw.2018.01.007

**Published:** 2018-01-31

**Authors:** F. Padrós, R. Knudsen, I. Blasco-Costa

**Affiliations:** a Fish Diseases Diagnostic Service, BAVE, Facultat de Veterinària, Universitat Autònoma de Barcelona, Bellaterra (Cerdanyola del Vallès), Barcelona, Spain; b Department of Arctic and Marine Biology, UiT The Arctic University of Norway, Langnes, P.O. Box 6050, 9037 Tromsø, Norway; c Natural History Museum of Geneva, PO Box 6434, CH-1211 Geneva 6, Switzerland

**Keywords:** Helminths, Eyeflukes, Retina, Pathology, Salmonidae, Freshwater

## Abstract

The eye represents an immune privileged organ where parasites can escape host reactions. This study provides the first systematic evidence of the pathology associated with *Diplostomum* sp. infection in the eye retina of fish (i.e. Arctic charr). Histological sections showed that the trematodes caused mechanical disengagement between the retinal pigmentary epithelium and the neurosensory retina, with damaged cones and rods in the outer segment and epithelium reduced to a single layer of pigmentary cells. The metacercariae were “floating” in possibly fluid-filled vesicles together with several round cells, mostly located in the anterio-dorsal and anterio-ventral areas of the eye near the iris. The round cells may indicate internal retinal damage repair mechanisms, without connections to the general immune system. Metacercariae intestines contained pigmented cellular debris indicating that they feed on retinal epithelium. These retinal lesions may have similar vision effects as focal retinal detachment in vertebrates. *Diplostomum* metacercaria alters fish visual acuity but may in a lesser degree lead to a severe or total visual impairment because of repairing mechanisms. The pathology in the retina seems thereby to be dependent on fish size, age and dose.

## Highlights

• Histological description of the distribution of *Diplostomum* in the eye of Arctic charr *Salvelinus alpinus*.
• Metacercaria specifically located in spaces formed between the retinal pigmentary epithelium and the neurosensory retina.
• Lesions display closer similarities with those observed in retinal detachment in other species.
• Changes in the frequency and distribution of the lesions between morphs are suggested.

## Graphical abstract

**Figure:**
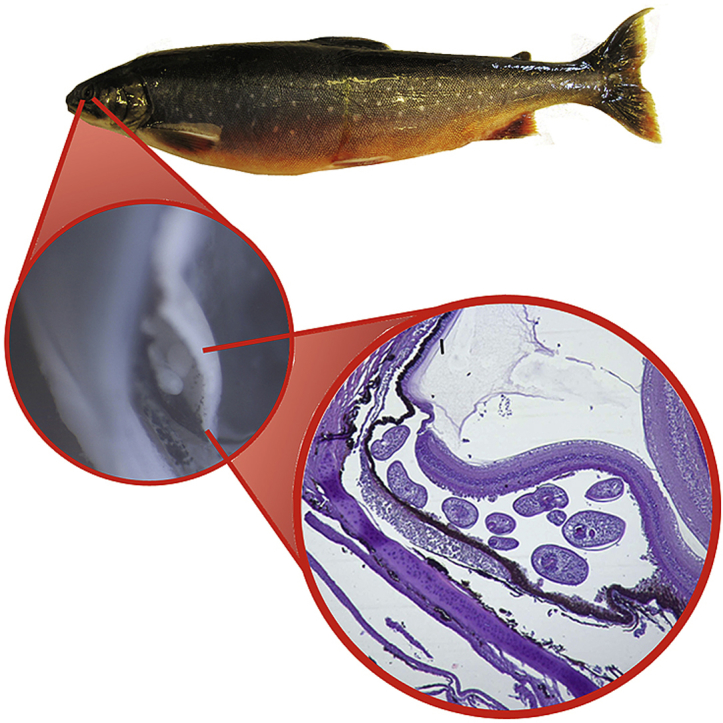

## 1 Introduction

The effect of parasites on their individual hosts and populations is known to have far reaching consequences for the whole ecosystem functioning (Poulin, 1999). There are multiple examples on how fish parasites can affect the survival or fitness of their host directly and indirectly (e.g. causing mechanical damage, modifying behaviour or personality traits, etc.; Poulin, 2010, Poulin et al., 2012, Kortet et al., 2010) and thereby influence ecological relationships within the ecosystems (e.g., Thomas et al., 1998, Marcogliese, 2004, Lefèvre et al., 2009). Most commonly, parasites exert sub-lethal chronic effects on their host reducing their resilience to the environment. Thus, evaluating the characteristics of the diseases, the pathologies associated to parasites and the general negative impact on their hosts are important topics not only in veterinary science but also for ecological and evolutionary research.

Vision is one of the most important sensory systems for many fish species (Bowmaker and Loew, 2008). The eyes display similar structure and function as terrestrial vertebrates although fish eyes can present a large diversity in adaptations to different environments and lifestyles. The role of the eyes and vision accuracy in the adaptation to the different environments is paramount for functions such as prey capture or predator detection. As in most vertebrate species, eye pathology represents an interesting research area due to the unique and complex anatomical and histological characteristics of the ocular structures and to the specific diseases and pathological conditions that can take place in this organ (Koppang and Bjerkås, 2006). Diseases affecting fish eyes have been widely described in the literature. The internal structure of the eye could represent an immune privileged structure (Caspi, 2013) and thereby parasites can escape reactions mobilized by the host to reduce negative effects induced by parasites. Amongst parasitic diseases in the eyes of fish, species of the trematode genus *Diplostomum* von Nordmann, 1832 (also known as eyeflukes) represent one of the most frequently reported (Chappell, 1967). *Diplostomum* spp. are obligate parasites of fish-eating birds, have three-host life cycles involving freshwater lymnaeid snails and fish as intermediate hosts and are widely distributed across the Holarctic. Metacercariae of *Diplostomum* spp. in the eye tend to be site-specific (Brady, 1989, Locke et al., 2010a, Blasco-Costa et al., 2014), restricted to the lens, vitreous humour or retina. Species infecting the eye lens are more closely related to each other than to species in other tissues (Blasco-Costa et al., 2014), and less host-specific than congeneric species infecting a different eye structure (Locke et al., 2010a, Locke et al., 2010b, Blasco-Costa et al., 2014). The low host-specificity of lens infecting species has been related to relatively low immune responses in this organ (Locke et al., 2010b, Locke et al., 2015).

Larval stages of *Diplostomum* spp. located in the eyes and brain of fish are considered major pathogens, causing variable fitness costs including reduced host survival (e.g. Crowden and Broom, 1980, Shigin, 1986, Chappell et al., 1994). The effects of lens infecting diplostomids have been reported widely, although few histopathological studies of the lens or other infected eye tissues are available (Williams, 1967, Chappell, 1967, Lester and Huizinga, 1977, Shariff et al., 1980, Grobbelaar et al., 2015, Stumbo and Poulin, 2016, Griffin et al., 2017). Typical alterations and lesions documented from eyefluke infection in the lens are exophthalmia, local haemorrhage, lens cataract, thickening or complete destruction of the lens, reduced fish growth, emaciation and deformities of the vertebral column. To the best of our knowledge however, only two studies have provided some information on the pathology associated to non-lens infecting *Diplostomum* spp. particularly those in the retina (Lester and Huizinga, 1977, Shariff et al., 1980). Diplostomids in the retina have been much less documented and studied than their congenerics in the eye lens of fish, mostly due to the difficulty of carrying out meticulous dissections of the eye to identify the precise site of infection.

*Diplostomum* spp. infecting the eye have been found in a large number of freshwater fish species belonging to phylogenetically distant orders, including those of economical importance (e.g., Anguilliformes, Clupeiformes, Cyprinodontiformes, Perciformes or Salmoniformes) (see e.g., Gibson, 1996 and references therein). Recent molecular studies have confirmed the presence of distinct lineages (putative species) of *Diplostomum* in the retina of Arctic charr (*Salvelinus alpinus*), brown trout (*Salmo trutta*), three-spines stickleback (*Gasterosteus aculeatus*) and European perch (*Perca fluviatilis*) (Blasco-Costa et al., 2014, Kuhn et al., 2015). The Arctic charr is one of the salmonids where *Diplostomum* spp. have been often reported and their effects studied under laboratory conditions (Frandsen et al., 1989, Knudsen, 1995, Skarstein et al., 2005, Voutilainen et al., 2009, Blasco-Costa et al., 2014). Wild populations of Arctic charr often split in different morphs (Jonsson and Jonsson, 2001, Klemetsen, 2010) as response to use of different habitats and/or feeding preferences that also results in differences in parasite fauna (e.g., Malmquist et al., 1992, Siwertsson et al., 2016). These different morphs present noticeable anatomical differences as putative ecological adaptations amongst which, eye size and position in the head are particularly relevant (Klemetsen et al., 2002, Skoglund et al., 2015). The deep-water morphs have relatively larger eyes (Skoglund et al., 2015) but their vision capabilities (photoreceptors) seem similar compared to their sympatric upper water morph (Kahilainen et al., 2016). These characteristics suggest that vision may be important for deep-water morphs. For instance, it may be likely involved in food-gathering and predator detection (Knudsen et al., 2016b). Thus, the impact of a specific parasitic infection such as *Diplostomum* spp. should be taken into account in the evaluation of the biological, ecological and evolutionary aspects of different Arctic charr morphs.

In this study, we focus on describing the pathology associated with infection by *Diplostomum* spp. in the eye retina of Arctic charr. We study fish from recently discovered polymorphic populations in Skogsfjordvatn, Northern Norway (Knudsen et al., 2016a). As no specific descriptions on the pathology in the retina has been published before in isolation of other infections in the eye, a detailed study on the main lesions and alterations caused by this trematode was considered necessary in order to provide a deeper knowledge on the potential damage in the visual capacities of these fishes. Furthermore, we evaluate whether parasite infections affect Arctic charr morphs differently and discuss briefly possible ecological consequences.

## 2 Materials and methods

### 2.1 Sampling

Skogsfjordvatn is an oligotrophic, dimictic coastal lake situated at Ringvassøy Island, in sub-Arctic Norway. The lake has an area of 13 km^2^, a maximum depth of about 100 m and is icebound for 6 months (December to May). Skogsfjordvatn is an open lake system with elevation of 20 m a.s.l., a 2 km long outlet river with migratory Atlantic salmon (*Salmo salar*), brown trout, Arctic charr and European eel (*Anguilla anguilla*). Additionally, there is freshwater resident brown trout, three-spined stickleback, and three Arctic charr morphs that differ in resource use, trophic morphology, parasite infection, life-history traits and are genetically differentiated (Skoglund et al., 2015, Knudsen et al., 2016a, Siwertsson et al., 2016, Simonsen et al., 2017, Smalås et al., 2017). The littoral spawning omnivore morph (LO-morph) utilize shallow water benthic and zooplankton prey resources. The two profundal morphs differentiate in resource use as the profundal spawning benthivore morph (PB-morph) is small sized and feeds on small deep-water benthic invertebrates, while the profundal spawning piscivore morph (PP-morph) mainly eat fish as prey (Knudsen et al., 2016b). Arctic charr is the numerically dominant species in the fish community and the only fish species inhabiting both the pelagic and profundal habitats.

Fish were sampled in late October and November 2015. We used multi-mesh benthic (1.5 m deep) gill-nets (5 m panels with mesh sizes: 5.5, 6, 8, 10, 12.5, 15, 18.5, 22, 26, 35, 45 mm, knot to knot) placed in the littoral zone (0–12 m depth) and the profundal zone (at 25–45 m depth). Fish length (fork-length) was measured (mm), and all Arctic charr were visually assigned to one of the three morphs, the LO-, the PB- and the PP-morphs (see Skoglund et al., 2015, Simonsen et al., 2017 for more details). For the present study, 16 LO-morph (mean: 243, range 171–324), 9 PB-morph (mean: 105, range 95–118) and 7 PP-morph (mean: 207, range 144–258) of Arctic charr were selected and processed for histopathology studies.

On the lake shore, Arctic charr eyes were injected through the sclera with Davidson's fixative solution (30mL95% ethyl alcohol, 20 mL 10% neutral buffered formalin, 10 mL glacial acetic acid, 30 mL distilled water; see Moore et al., 1953). Immediately after, the head of half the specimens of each morph was dislodge and placed in plastic containers submerged in the fixative solution, while in the other half only the eyes were dislodge and transfer into small containers with the fixative. After 24 h, the tissue was removed from the fixative, rinsed with tap water and put into 10% neutral buffered formalin for storage at room temperature until use.

### 2.2 Histological processing

For each fish sample, one of the eyes was selected and longitudinal and transversal axes were measured before processing. A sagital section including the optic nerve was performed and lens was removed. After section, some samples were examined in Petri dishes covered with saline solution under the binocular microscope. Left and right half eyes and lens were put in histological cassettes and processed separately in paraffin according to standard techniques. Three different sections (4 μm) at different levels were performed from each half eye. Sections were stained with Haematoxylin and Eosin and mounted in DPX.

## 3 Results

**Fig. 1:**
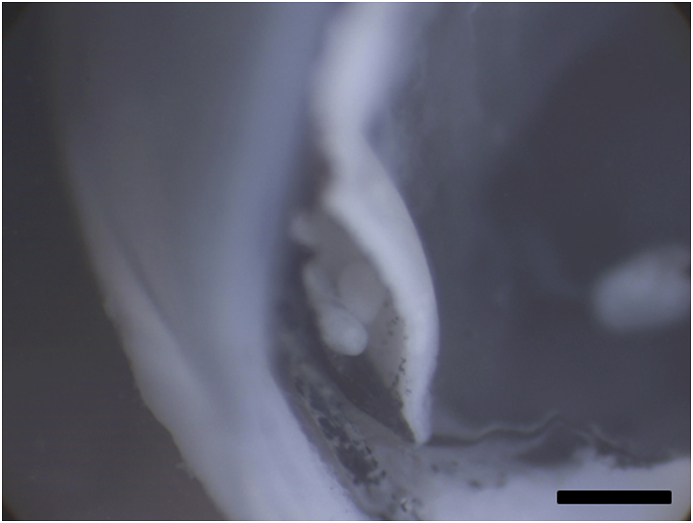
Section of the fixed eyes observed under the stereomicroscope. Notice the presence of large vesicles inside the retina with several *Diplostomum* specimens. Scale bar = 1 mm.

**Fig. 2:**
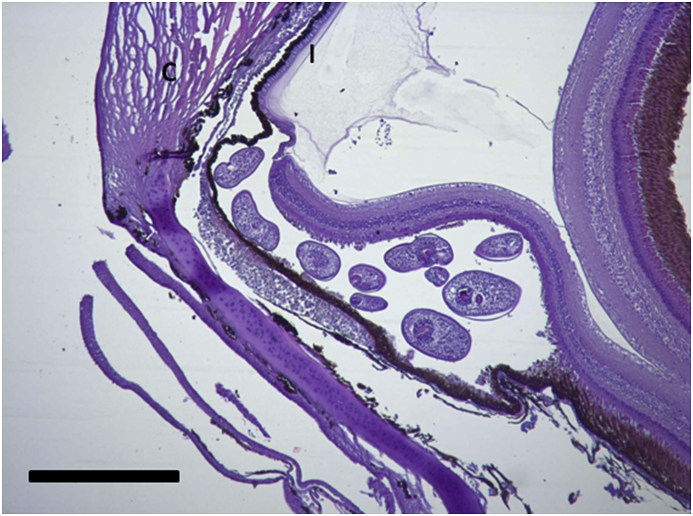
Vesicle with several *Diplostomum* specimens in a histological section. Vesicles are typically located near the ciliary body/retina contact area. C: cornea. I: iris. H/E. Scale bar = 1 mm.

Histological sections revealed the presence of digenean metacercariae in most of the processed samples and in all three morphs (prevalence in LO: 87.5%, PP: 100% and PB: 100%, as detected in sections). Preliminary data confirmed the metacercariae in the retina of Arctic charr from this lake correspond to a single species of *Diplostomum*, with 100% prevalence in all three morphs (unpublished, Blasco-Costa and Knudsen). Metacercaria were observed exclusively within the retina and no metacercaria were detected in other locations in the eye. After sectioning, 0.5–1 mm single vesicles within the retina and between the pigmentary layer and the inner retinal layers could be observed under the binocular microscope at low magnifications (Fig. 1). In most cases it was possible to detect many metacercariae clustered inside a section of these vesicles. Histological sections confirmed the presence of these large vesicles with several metacercaria inside (Fig. 2). These vesicles were mostly allocated in the anterio-dorsal and anterio-ventral areas of the eye, close to the transition of the retina to the ciliary body and iris. The number of parasites observed in each section was particularly high (up to 7–10 metacercariae) in most of the LO-morphs with detected parasites in the eye although vesicles with 2–4 parasites could also be observed in the PP- and the PB-morphs.

**Fig. 3:**
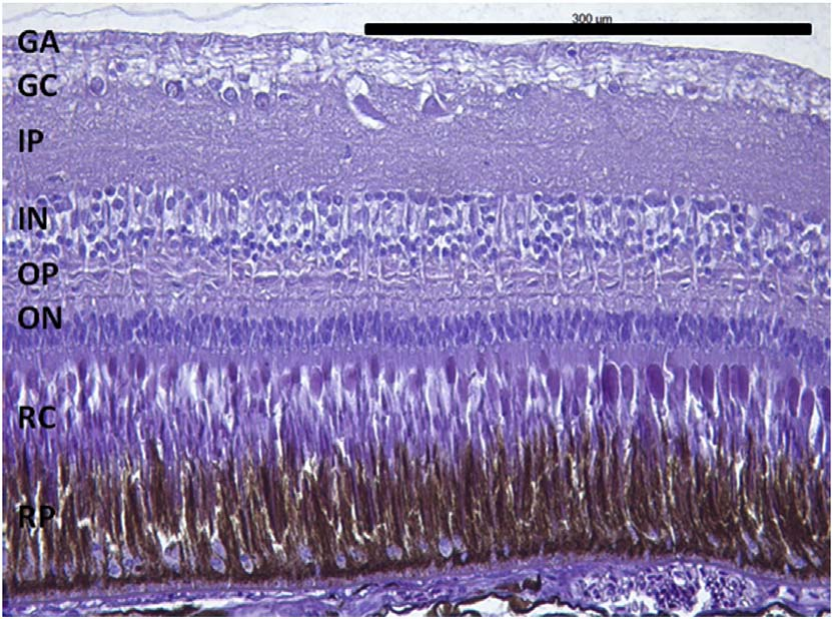
Normal aspect of the retina in Arctic charr with different layers. From the eye exterior to the eye interior: (RP): Retinal pigment epithelium. (RC) Cones and rods layer. (ON) Outer nuclear layer. (OP) Outer plexiform layer. (IN) Inner nuclear layer (IP) Inner plexiform layer. (GC) Ganglion cell layer. (GA) Axons of the ganglion layer. Scale bar = 300 μm.

**Fig. 4:**
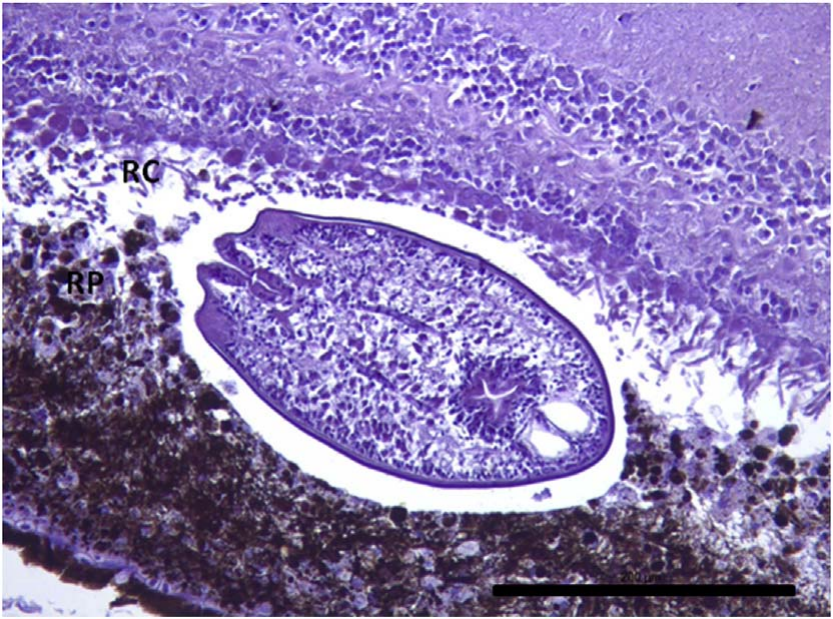
*Diplostomum* sp. metacercaria within the retinal structures. This specimen is clearly placed between the retinal pigmented epithelium (RP) and rod and cones layer (RC) creating a small space between them and the parasite. Damaged retinal pigment epithelium is clearly observed and also rod and cone layer display morphological alterations. Scale bar = 200 μm.

**Fig. 5:**
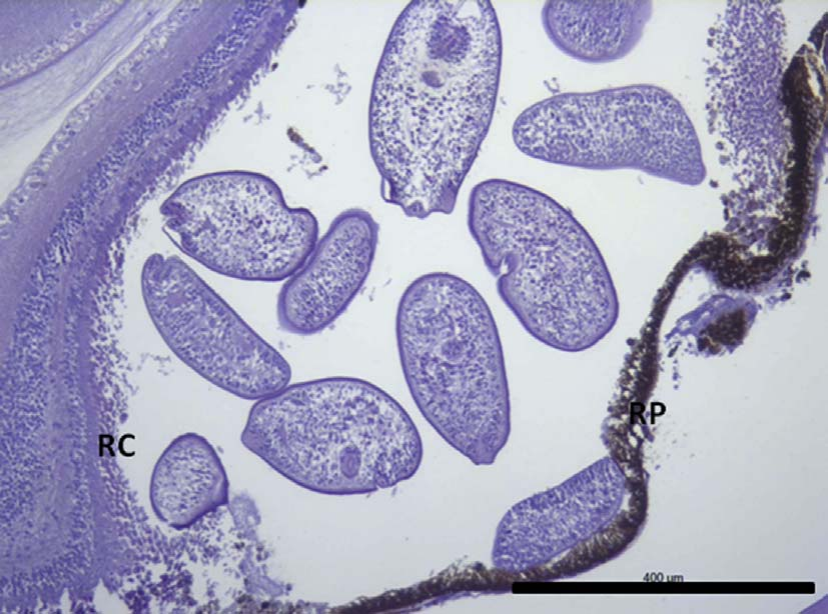
Large vesicle with sections of many *Diplostomum* specimens. The vesicle clearly creates a large space between RP and RC. Scale bar = 400 μm.

**Fig. 6:**
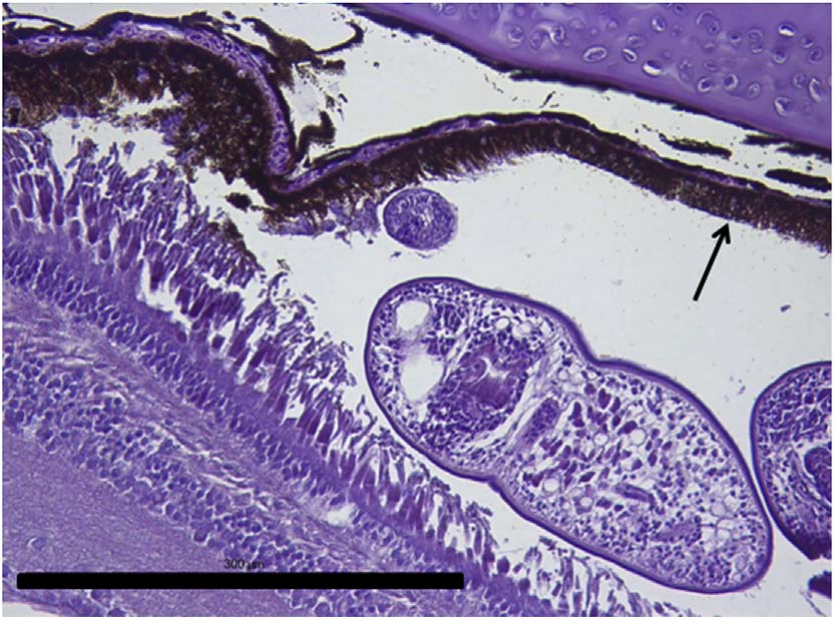
Edge of one of the vesicles produced by the accumulation of parasites. Retinal pigment layer and rods and cones layer display a progressive alteration in their structure and finally both layers become detached. Notice the reduction of the thickness of the RPE (arrow) in the vesicle. Scale bar = 300 μm.

**Fig. 7:**
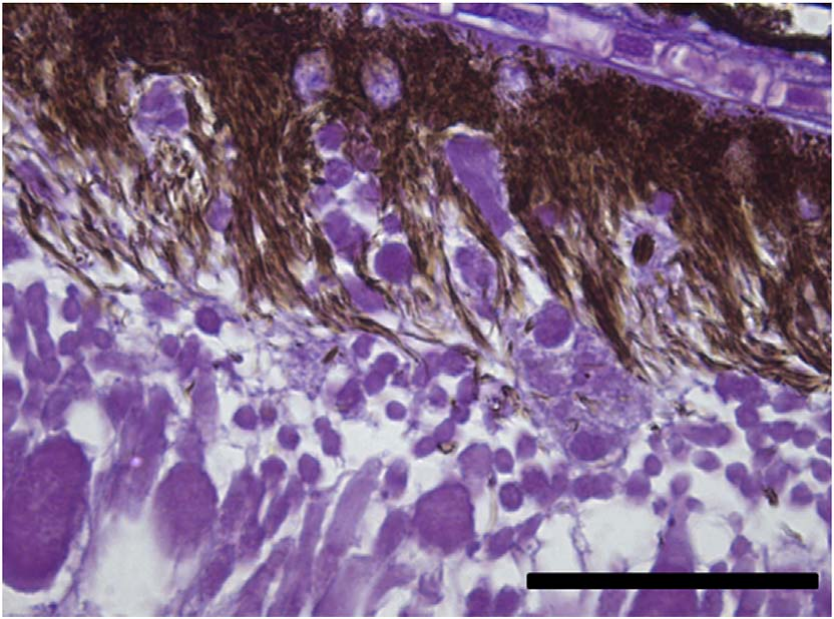
Early lesions in RPE and RC in the retina closer to the edge of the vesicles. Cones and rods display a disorganized pattern between the pigmented processes of the RPE. Scale bar = 100 μm.

**Fig. 8:**
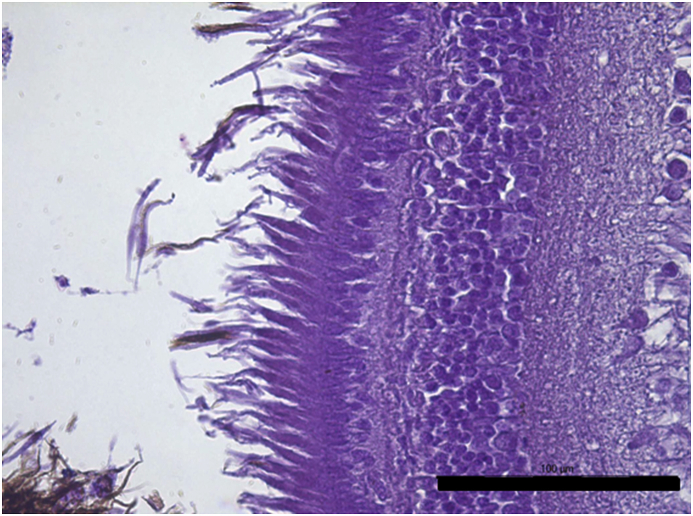
Cones and rods ripped out and completely detached from the pigmented epithelium. Scale bar = 100 μm.

**Fig. 9:**
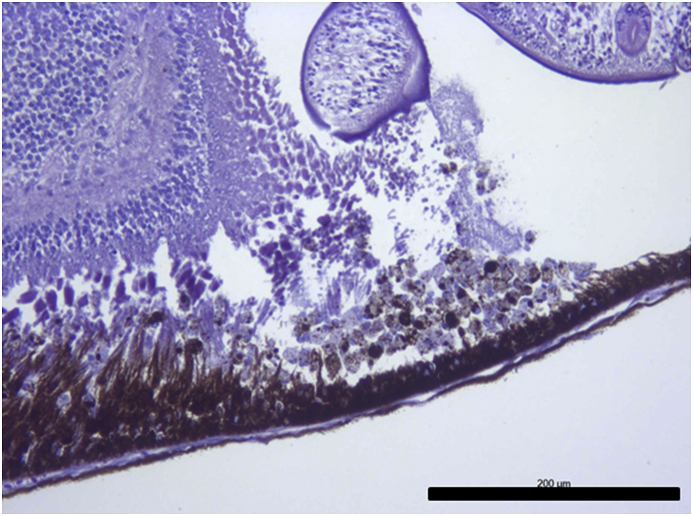
Changes in the RPE associated to the presence of parasites and vesicle generation. Notice the presence of a large number of cells with round to oval shape. Scale bar = 200 μm.

**Fig. 10:**
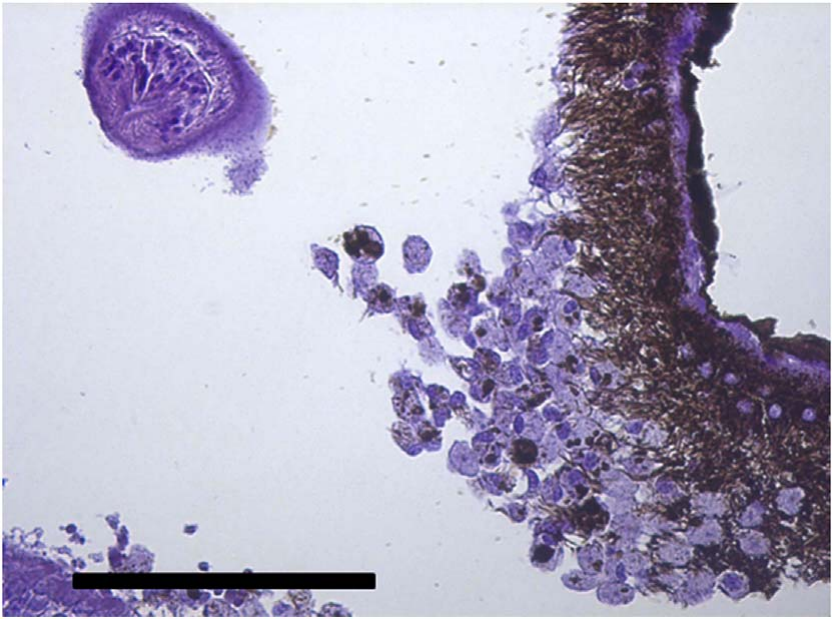
Round-oval cells and cytoplasm filled with melanin pigment and large pale-blue granules. Scale bar = 100 μm. (For interpretation of the references to colour in this figure legend, the reader is referred to the Web version of this article.)

Vesicles and parasites were always detected between the retinal pigmentary epithelium (RPE) and the neurosensory retina (NSR). In Arctic charr, as well as in other fish species, the outermost retinal layers are formed by epithelial cells with large amounts of pigments arranged on a thin membrane resembling Bruch's membrane (Fig. 3). The RPE in fish is usually thicker than in most terrestrial vertebrates. Retinal pigmentary epithelial cells display large cytoplasmatic projections towards the photoreceptor projections layer (rods and cones) and intermingling these structures. The presence of these parasites seems to produce a disengagement of these two structures and creating empty spaces (Fig. 4) or vesicles. In each vesicle, the central space was occupied by metacercariae “floating” in a fluid-filled space (Fig. 5) and sometimes a diffuse amorphous material was observed within the vesicles. The RPE in these vesicles was usually altered and limited to a single layer of pigmentary cells (Fig. 6). The opposite side of these vesicles was mainly formed by disorganized, damaged or destroyed cones and rods in the outer segment. However, the outer epithelial nuclear layer usually did not display signs of alteration. In the edge of these vesicles, the transitional zone between the pigmentary epithelium and the neurosensory retina was progressively altered (Fig. 7) and ripped up during the vesicle formation process (Fig. 8). One of the main changes observed is the presence of round to oval cells, with a single nucleus and cytoplasm filled with large pale-blue granules and melanin pigment in some cases (Fig. 9, Fig. 10). These cells are usually not observed in normal retina.

**Fig. 11:**
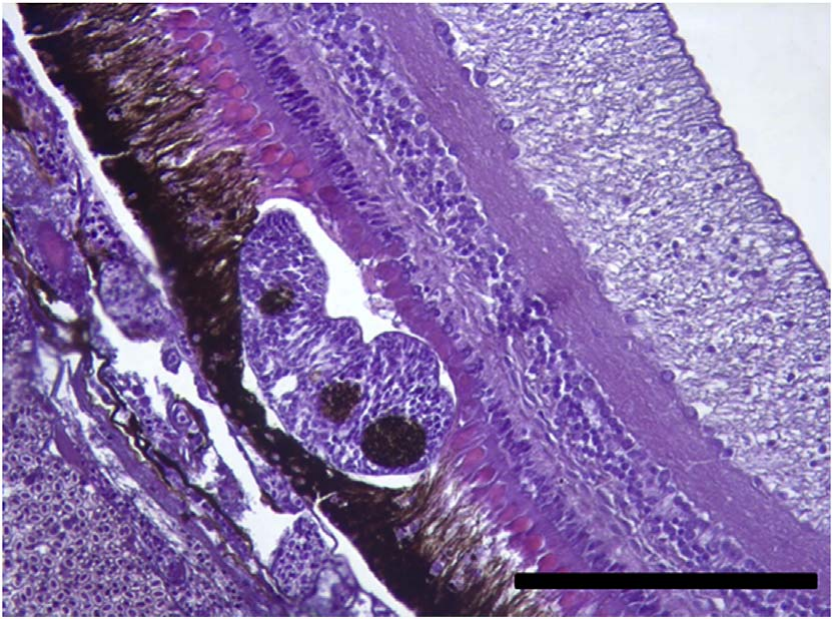
A single *Diplostomum* sp. metacercaria within the posterior retina with scarce development of surrounding vesicle and mechanical compression against the RPE and the cones and rods layers. Scale bar = 200 μm.

**Fig. 12:**
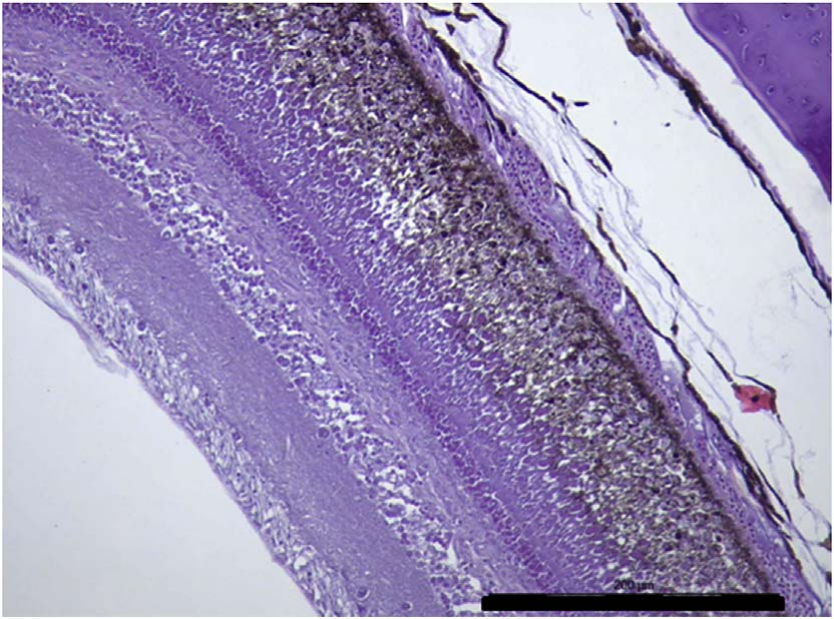
PP-morphs. Diffuse changes in the posterior retina affecting mainly the RPE layer suggesting potential healing. Scale bar = 200 μm.

Histological sections also revealed the presence of single metacercaria within the posterior retina in 10.3% of the fish but, in these cases with scarce or no vesicle development. Such alterations may vary from simple mechanical compression in the RPE and the cones and rods layer due to the presence of the parasite (Fig. 11), to more extensive lesions with partial disintegration of these two layers. Single metacercaria and related lesions were mainly found in the posterior part of the retina. Only in two individual PP-morphs the posterior retina displayed diffuse changes that could resemble healing of former lesions (Fig. 12), but in these cases no vesicles were formed. In these instances, although RPE and NSR were in close contact and no spaces were found, not all cones and rods were completely aligned in a perpendicular disposition and also some RPE cells with round shape were still present. No clear inflammatory response in terms of presence of inflammatory cells or haemorrhages was noticed in any of the lesions observed.

**Fig. 13:**
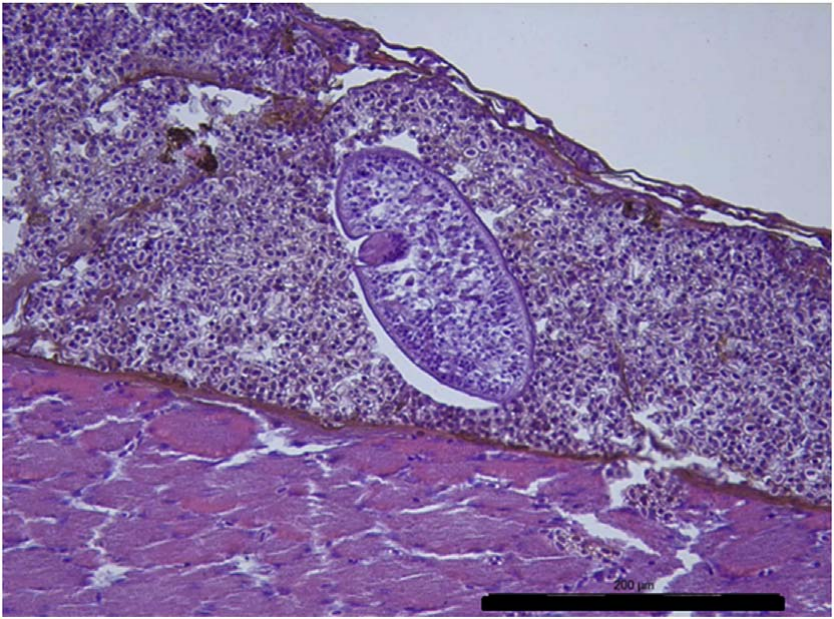
*Diplostomum* sp. metacercaria in a choroidal vessel. Scale bar = 200 μm.

Parasites localized in the different sections were easily recognized as metacercaria. In each vesicle, they were observed as “floating” organisms within a fluid-filled empty space. Typical structures recognized, such as oral sucker, pseudosuckers, intestinal caeca and also digested material were commonly seen within the metacercaria (Fig. 4, Fig. 5, Fig. 6). In many cases, material from the retina represented by pigmented material very similar to cellular debris from the RPE was observed within the metacercariae, as well as some small dilatations with diffuse amorphous material very similar to the material observed within the vesicles (Fig. 10, Fig. 11). In one of the samples, a single metacercaria was seen within one choroidal vessel (Fig. 13).

## 4 Discussion

In this study, retinal damage by *Diplostomum* sp. eye infection has been detected and confirmed in all Arctic charr morphs from Lake Skogsfjordvatn suggesting a potential impairment of the vision of these fishes. The histopathological changes in the retina associated to infection of *Diplostomum* sp. in Arctic charr indicates that metacercaria display a specific tropism towards the transition zone between the neurosensory retina (NSR) and the retinal pigmentary epithelium (RPE). A similar specific tropism to this zone was described in *Perca flavescens* infected by *Diplostomum adamsi* (Lester and Huizinga, 1977) and also in rainbow trout (Shariff et al., 1980). In the latter study, degenerative changes in the retina were accompanied with strong alterations in the lens. The presence of metacercaria in this transition zone generates mechanical disengagement of the RPE from the NSR, as this junction is anatomically the weakest zone in the retina and no junctions are formed between these two layers (Ghazi and Green, 2002). Changes induced by the presence of the parasites are limited to the NSR and RPE. Once detached from the RPE, rods and cones display progressive changes, with some of them destroyed and disconnected from the NSR. In most sections, the external molecular layer was apparently non-affected. These observations could relate to a high turnover and regeneration capacity of this retinal layer and might suggest a transitional reduction of the visual capacity due to these parasites and to a lesser degree lead to permanent visual impairment.

After detachment, RPE also displays major changes represented by the presence of round to oval cells. These cells probably represent retinal pigmentary epithelial cells disconnected from the rest of cells. RPE in fish is a relatively thick layer compared with terrestrial vertebrate species and represents approximately 20% of the total retinal thickness. It is formed by columnar epithelial cells with large cytoplasmic processes and with melanosomes with a high capacity for light-dependent migration towards NSR structures (King-Smith, 2016). The alteration of the RPE by the parasites therefore seems to induce the generation of a significant number of these round cells. Most of these cells do not display signs of apoptosis or necrosis and can also be found in large numbers in sections where retinal repair is observed. These cells display a large number of granules inside their cytoplasm resembling phagosomes. Continuous phagocytic capacity in the RPE of photoreceptor outer segment membranes damaged by photo-oxidative stress has been demonstrated in vertebrates (Strauss, 2012). Moreover, modified RPE cells have been described displaying phagocytic activity in several diseases affecting the fish retina (Koppang and Bjerkås, 2006). The present observations suggest that these detached RPE cells may act as macrophage-like in the repair processes within the retina, removing the cellular debris from RPE and NSR originated by the mechanical action of the parasites. No inflammatory cells were found in these lesions supporting that the fish retina and also internal parts of the eye are considered as an immune privileged tissues (Caspi, 2013). All the above together indicates that these changes should be considered as internal retinal damage repair mechanisms without connection with the general immune system. The cellular responses in the present case are very different to the response observed in infections associated with other *Diplostomum* spp. such as the response against *Diplostomum phoxini* in the brain tissue of the European minnow (Dezfuli et al., 2007).

This repair/regeneration process has also been claimed to operate as a basis for the lesions associated to retinal detachment in humans and other vertebrate species. The special features observed in the present case are unique and seem different as described in the retinal detachment pathogenesis (e.g., Ghazi and Green, 2002). These observations strongly suggest that *Diplostomum* sp. infection in the retina of Arctic charr may induce similar effects in the vision of the infected fish as focal retinal detachment in humans. As vesicles are mainly observed close to the iris, taking into account the relevance of the outlying retina for the peripheral vision, it can be suggested that retinal infection by *Diplostomum* sp. can alter the capacity of the fish to detect and respond to predators. Moreover, most of the new retinal cells in fish eyes are generated in the margin of the retina, in a region called the peripheral growing zone (Jimeno et al., 2003). Thereby a damage in these areas like in the present description, may also lead to a reduced capacity of regenerate the retina. Thus, an experimental following up study should be done testing potential dose-dependent effects and anti-predator responses to get more conclusive results. Parasite induced lesions in the central posterior retina seem to be focal and limited in extension, so they may represent a lesser problem for the fovea (i.e. sharp central vision). In any case, for diagnostic purposes, it is important to take into account that histological artefacts sometimes may have the same aspect as retinal detachments and should not be confused by real lesions induced by parasites. In such cases (artefacts), no changes in RPE or NSR cells are found.

Immunoprivilege of the eye chamber is a unique feature in the vertebrate organs and it is related to the particular anatomical characteristics of the eye, although in cases of severe damage in the eye, extensive inflammatory responses can be triggered. In case of low or mild internal damage, the absence of inflammatory response allows the fish eye to keep some of their functionality and may also promote a faster repair. In the present case, retinal repair uses the same strategies and mechanisms as the retina normally displays to eliminate and renew photoreceptor outer segments constantly altered due to continuous photo-oxidative damage. Therefore, it seems that retinal regeneration may develop relatively fast and minimizing the losses in visual acuity. RPE detached cells do not apparently display any response towards metacercaria. All these observations suggests an important specific adaptive component between these parasites and their hosts.

In our study system, metacercariae specifically enters the retinal structures and progresses through the RPE-NSR virtual layer until they reach the anterior part of the retina where they accumulate forming the vesicles previously described. No references on the specific entrance site into the eye exist for *Diplostomum* spp. In the case of *Diplostomum spathaceum*, the eye lens are achieved after migration of the diplostomula through blood vessels (Haas et al., 2007). In the present study it was possible to detect a single metacercaria within a blood vessel close to the choroides, which suggests that this could be the potential site of entrance, including posterior choroidal vessels but also blood vessels presents in the iris. In addition, no parasites were found in the vitreous humour nor migrating parasites or induced lesions were found in external retinal layers.

Vesicles or blister-like lesions where parasites are found aggregated can be associated to enlarged spaces filled with liquids. Retina plays a major role in the transport of ions and nutrients from the choroid, so liquid accumulation within these vesicles can be expected. Large numbers of parasites can be seen ‘floating’ in these spaces suggesting a suitable microhabitat for *Diplostomum* sp. metacercariae. This appropriate microhabitat can be related to the immunoprivileged area and also to the presence of nutritional resources (e.g., retinal cell structures). Groups of melanosomes are frequently observed inside the digestive tube of the metacercaria and also in some cases vesicles with amorphous material was observed within the parasite bodies. These observations suggest actively feeding of the metacercaria on intertissular fluids, on cellular debris from damaged RPE and probably NSR. Active feeding has been previously proposed in other unencysted metacercariae of *Diplostomum* spp. (Blasco-Costa et al., 2014, Faltýnková et al., 2014), particularly at the early stages of the metacercaria development within the fish eye (Podvyaznaya, 1999). As RPE is considered a tissue particularly rich in vitamin A (Zhong et al., 2012) active feeding on this cellular debris could represent a special implication or requirement of vitamin A in metacercarial metabolism. Haas et al. (2007) found that migrating *Diplostomum* cercariae used melatonin (produced in the fish eye retina) as cue to reach the eye among several other compounds tested. Since Vitamin A is abundant in the retina, this compound could also work as a target for orientation within the fish host for *Diplostomum* spp. cercariae.

The proposed moderate impact of parasites on the retina suggests that the pathology thereby seem to be dependent on several factors. First, the size of the fish as smaller fish will have smaller eyes/habitats. Second, the age of the fish as metacercariae generally accumulate over the life span of the host (no sign of parasite death has been observed in our samples). The damages are also highly dose-dependent and therefore will be accentuated with a persistent high infection pressure over time. Fish seem generally to suffer from diplostomiasis as highly infected individuals have lower capture rate of prey compared with less infected fish (e.g. Crowden and Broom, 1980). Thus, the ecological consequences for the individual host could be severe in early and late stages and in systems with high infection pressure.

The three morphs present in our study, have relevant differences in head and eye morphology as well as important ecological adaptations (Skoglund et al., 2015). The upper-water (LO-morph) and the deep-water PB-morphs from sub-Arctic clear water lakes have relatively similar visual capabilities and pigments (photoreceptors) (Kahilainen et al., 2016), suggesting that the parasite will have relative similar effects in their different hosts. However, although parasites and similar lesions have been found in all three morphs, some differences in the aspect and distribution of the changes associated to the parasites have been observed in the present study. The LO-morph tends to present larger vesicles filled apparently with a higher number of parasites (75% of the LO-morph specimens) and in some PP-specimens (42.9%) more extensive parasite induced changes were seen in the posterior retina. Thus, these extensive repairs observed in the piscivore PP-morph could indicate that this morph is highly dependent on their vision capabilities to e.g. catch prey (small fish) in dark environments (deep-water). Furthermore, as the small-sized benthivore PB-morph have smaller eyes than their sympatric LO- and PP-morphs, therefore they could be more severally affected by *Diplostomum* sp. infections. The PB-morph has to detect small, sediment buried prey in addition to spot predators visually at deep-waters (Knudsen et al., 2016a). These differences suggest that the retinal pathology observed may influence upon the relevance on visual acuity and prey/predator detection. A recent study partly supported such idea. Kortet et al. (2017) detected maternal inherited differences in vulnerability to eye-fluke parasites that were associated to host behavioural activity. This could lead to selection for increased vulnerability to parasitism and intensified predation risk (Kortet et al., 2017), in particular between sympatric Arctic charr morphs that are genetically different and reproductively isolated (Simonsen et al., 2017, Smalås et al., 2017). These hypotheses deserve further investigation by doing field surveys and experimental studies on *Diplostomum*-host interactions.

To sum up, in this paper we describe specific interactions and pathological events established between *Diplostomum* larvae and the retina of Arctic charr at the histological level. This observation suggests a unique and specific placement of the parasites within the retina, causing damages similar to the retinal detachment in other vertebrate species but also suggesting a particular benefit for the parasite. The lesions caused by the parasite in the eye retina of the fish suggest that visual capacity can be impaired. Furthermore, the presence and also the absence of severe damages or responses such as inflammation suggests that retinal structures are constantly regenerating from parasites action, but implies an additional physiological cost of infection for the host.

## Conflicts of interest

None.
